# 1-3-7 surveillance and response approach in malaria elimination: China’s practice and global adaptions

**DOI:** 10.1186/s12936-023-04580-9

**Published:** 2023-05-09

**Authors:** Boyu Yi, Li Zhang, Jianhai Yin, Shuisen Zhou, Zhigui Xia

**Affiliations:** National Institute of Parasitic Diseases, Chinese Center for Disease Control and Prevention (Chinese Center for Tropical Diseases Research), NHC Key Laboratory of Parasite and Vector Biology, WHO Collaborating Center for Tropical Diseases, National Center for International Research on Tropical Diseases, Shanghai, 200025 China

**Keywords:** Malaria elimination, 1-3-7 approach, Surveillance and response, Popularization

## Abstract

There has been a significant reduction in malaria morbidity and mortality worldwide from 2000 to 2019. However, the incidence and mortality increased again in 2020 due to the disruption to services during the COVID-19 pandemic. Surveillance to reduce the burden of malaria, eliminate the disease and prevent its retransmission is, therefore, crucial. The 1-3-7 approach proposed by China has played an important role in eliminating malaria, which has been internationally popularized and adopted in some countries to help eliminate malaria. This review summarizes the experience and lessons of 1-3-7 approach in China and its application in other malaria-endemic countries, so as to provide references for its role in eliminating malaria and preventing retransmission. This approach needs to be tailored and adapted according to the region condition, considering the completion, timeliness and limitation of case-based reactive surveillance and response. It is very important to popularize malaria knowledge, train staff, improve the capacity of health centres and monitor high-risk groups to improve the performance in eliminating settings. After all, remaining vigilance in detecting malaria cases and optimizing surveillance and response systems are critical to achieving and sustaining malaria elimination.

## Background

Malaria is one of the world’s most common and serious tropical diseases. There were an estimated 241 million malaria cases in 2020 in 85 malaria endemic countries. Although malaria case incidence (i.e. cases per 1000 population at risk) reduced from 81 in 2000 to 59 in 2020, and malaria deaths reduced from 896,000 in 2000 to 627,000 in 2020, the current level is still unacceptably high and the burden of malaria remains severe [1]. In 2020, malaria case incidence and deaths increased again compared with 2019 due to service disruptions during the COVID-19 pandemic.

To accelerate progress towards malaria elimination, there is an urgent need to implement the strategies recommended by the World Health Organization (WHO) to reduce malaria morbidity and mortality. A pillar of the Global Malaria Technology Strategy 2016–2030 is to transform malaria surveillance into a key intervention [2]. The goal of surveillance is to reduce the burden of malaria, eliminate the disease and prevent its retransmission. The 1-3-7 surveillance and response approach proposed by China plays a key role in the progress of malaria elimination in China. The WHO also recognized a strict adherence to the timelines of 1-3-7 approach as critical to malaria elimination [3]. Therefore, this approach was popularized by the WHO to be a guideline to instruct malaria control programmes globally, especially in countries where malaria is close to elimination [4].

Due to large-scale malaria control efforts, the number of malaria cases in China dropped from an estimated more than 30 million before 1949 to 14,491 in 2009 [5, 6]. Given this progress in malaria control, China proposed the National Malaria Elimination Action Programme (NMEAP) in 2010, aiming to eliminate malaria nationwide by 2020 [7]. To support this goal, the 1-3-7 approach was developed and scaled up in 2011 as a technical specification [8]. The last indigenous case was reported in Yunnan Province in April 2016, and no indigenous cases have been reported since 2017 [9]. In 2021, the WHO certified China as malaria-free country. The transformation of China from a malaria-endemic country to a malaria-free country has played an important role in the development of China’s public health and the global malaria eradication. The 1-3-7 approach has played an important role in eliminating malaria in China, which detects cases early, investigates them and rapidly responds to prevent the spread of the disease. As a result, the 1-3-7 approach has been popularized and adopted in some countries to help eliminate malaria [4, 10].

This review summarizes the experience and lessons of 1-3-7 approach in China and its application in other malaria-endemic countries and regions, so as to provide references for its role in eliminating malaria and preventing retransmission.

## Methods

**Fig. 1:**
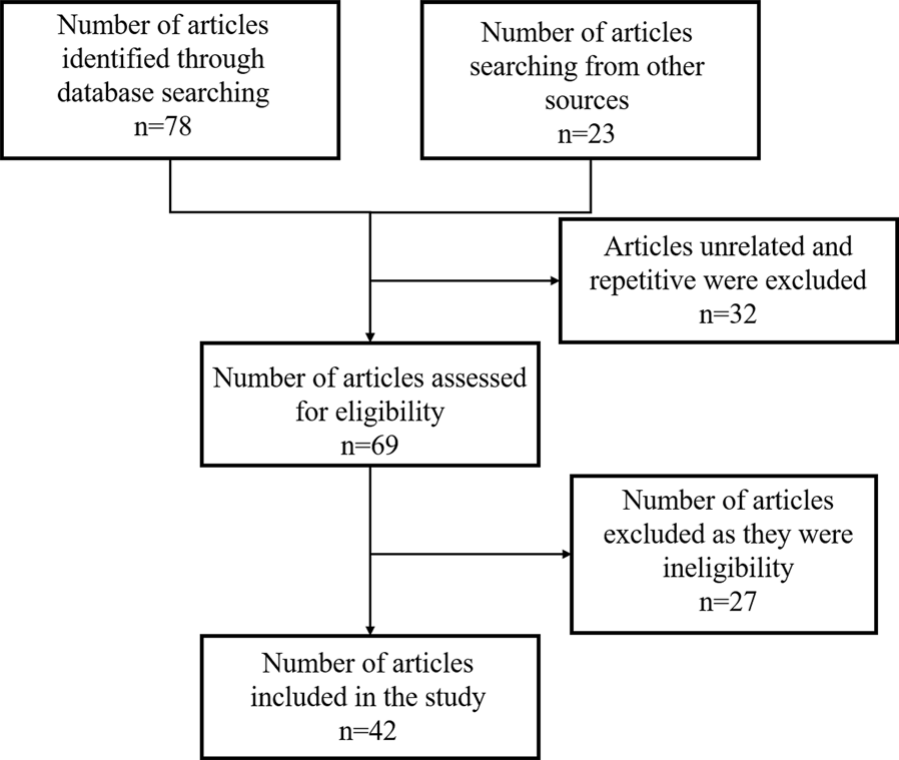
Article selection strategy

A search using the key words “Malaria” and “1-3-7” was carried out in PubMed and Web of Science for studies published until 1st May 2022. Significant efforts were made to search grey literature sources, mainly available from the Chinese Center for Disease Control and Prevention. After reviewing the title and abstract of the articles, all unrelated and repetitive articles were excluded from the review. The remaining paper were evaluated for full text review to decide whether it should be chosen for inclusion. The inclusion criteria for selection of articles for review included: (1) how 1-3-7 approach is implemented or adapted; (2) challenges faced in 1-3-7 approach implementation and adaption; (3) suggestions on improving effectiveness of 1-3-7 approach (Fig. 1).

## Results

### The definition and content of 1-3-7 approach

**Fig. 2:**
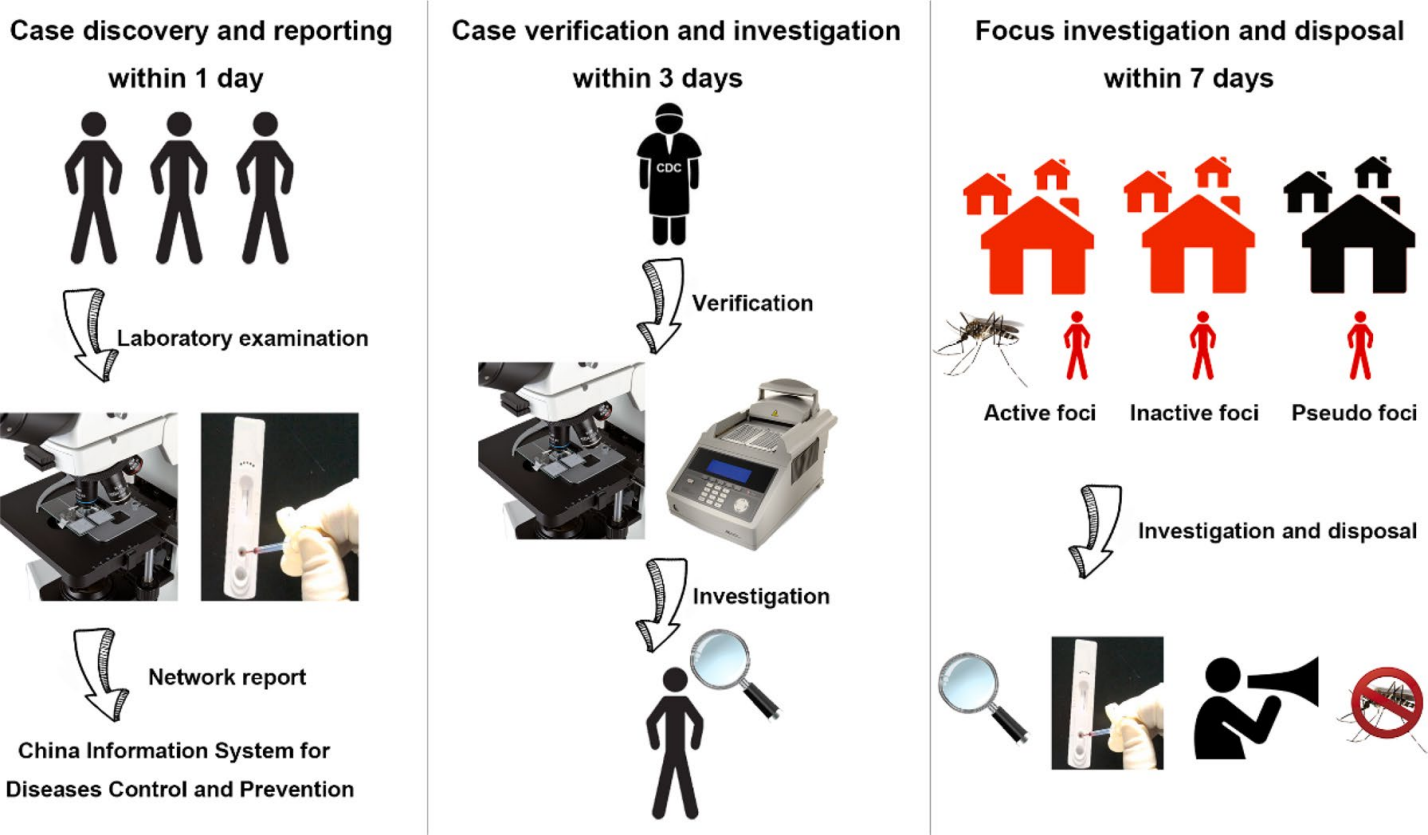
The flow chart of 1-3-7 strategy for malaria elimination

The guideline of 1-3-7 approach is to report cases within 1 day of diagnosis, complete case investigation within 3 days after reporting, and finish foci investigation and response within 7 days. The detailed processes are as follows (Fig. 2).

### Case reporting within 1 day

The clinician should make a preliminary diagnosis based on the patient’s epidemiological history and clinical manifestations. Any suspected, clinically diagnosed malaria cases and fever patients of unknown origin (FUO) should be promptly subjected to malaria-related laboratory examination [11]. More importantly, the health facilities should report the cases online directly through China Information System for Diseases Control and Prevention (CISDCP) within 24 h once they find them. If these institutions are unable to perform malaria-related laboratory examination or direct online reporting, they should seek help from superior institutions.

### Case verification and investigation within 3 days

Once cases are reported, the staffs of the disease prevention and control agency must immediately contact the reporting institutions and review the blood smear by microscopy of the reported cases. At the same time, professionals complete an epidemiological case investigation within 3 days. It is worth mentioning that those cases with negative blood smears diagnosed by counties (cities or districts) were further confirmed by PCR in superior provincial agency. For provincial disease prevention and control agency that are not currently qualified for PCR, the National Institute of Parasitic Diseases of Chinese Center for Disease Control and Prevention (CDC) guide or assist in the confirmation of cases.

### Focus investigation and response within 7 days

“Focus” refers to the villages, settlements or construction sites where malaria cases occur, which are divided into active foci (with transmission), inactive foci (with potential for transmission) and pseudo foci (without potential for transmission). Active foci are endemic areas in which indigenous malaria cases occur. Inactive foci refer to malaria cases occur in endemic areas outside transmission season. Or malaria cases occur during the transmission season, while there is no effective malaria vector (*Anopheles sinensis* cannot transmit *Plasmodium falciparum* in China). Pseudo foci mean malaria cases occur in non-malaria-endemic areas where there is a lack of vectors to cause malaria transmission. Given the risk of transmission in both active and inactive foci, disease prevention and control agency should organize investigation and response of foci within 7 days after direct online reporting of cases. The purpose of foci investigation is to assess the potential transmission risk. The investigation of foci includes the basic information of the endemic areas, population of *Anopheles*, and reactive case detection (RACD) such as microscopy or RDT of blood samples collected from people by house-to-house visits. The response of foci includes health education, vector control and treatment to eliminate possible sources of infection depending on transmission.

### Why it is “1-3-7” ?

A key process in malaria transmission is the maturation of male and female gametocytes in human blood. *Plasmodium vivax* develops gametocytes from sporozoites most rapidly, and its transmission can occur even before symptoms appear [12]. Malaria cases are, therefore, required to be reported and treated within 24 h. The aim is to stop the risk of malaria transmission in the first place. In consideration of the difficulty of case verification and investigation in different regions, it should be completed within three days. According to the parasite’s biological life cycle, *Plasmodium vivax* takes about 10–18 days to finish sporogonic cycle in a mosquito [13]. Considering that the average time for patients to seek treatment after onset is 3 days, focus investigation and response within 7 days can interrupt the transmission vector in time to eliminate malaria transmission. In addition, it takes 6 or 7 days from the time the *P. vivax* sporozoite enters the blood with the saliva of *Anopheles* mosquitoes to the completion of the exocytic phase. Then, after 48 h of development inside the erythrocyte, partial merozoites differentiates into male and female gametocytes [14]. It may only take 8 days to infect mosquitoes and cause malaria transmission. Given that a patient infected with *P. vivax* is already infectious at the time of onset, it is assumed that secondary infection has occurred. Focus investigation and response within 7 days can promptly detect secondary malaria and stop the further transmission of malaria.

### The timeliness and effectiveness of 1-3-7 approach in China

China proposed NMEAP in 2010, aiming to eliminate malaria in most regions by 2015 and eliminate it nationwide by 2020 [7]. The 1-3-7 malaria elimination approach was initially developed and rolled out in China in September 2011 [8]. The core of the 1-3-7 approach is timeliness, with strict reporting and response deadlines, to implement robust malaria elimination programmes to interrupt the transmission and prevent re-establishment. The completion of 1-3-7 approach has also been used as a core indicator to evaluate the quality of malaria elimination for all provincial administrative divisions in China. Between 2013 and 2020, all malaria cases notified were reported within 1 day, 94.5% were verified and investigated within 3 days after diagnosis, and 93.4% of foci finished investigation and response within 7 days [15].

**Fig. 3:**
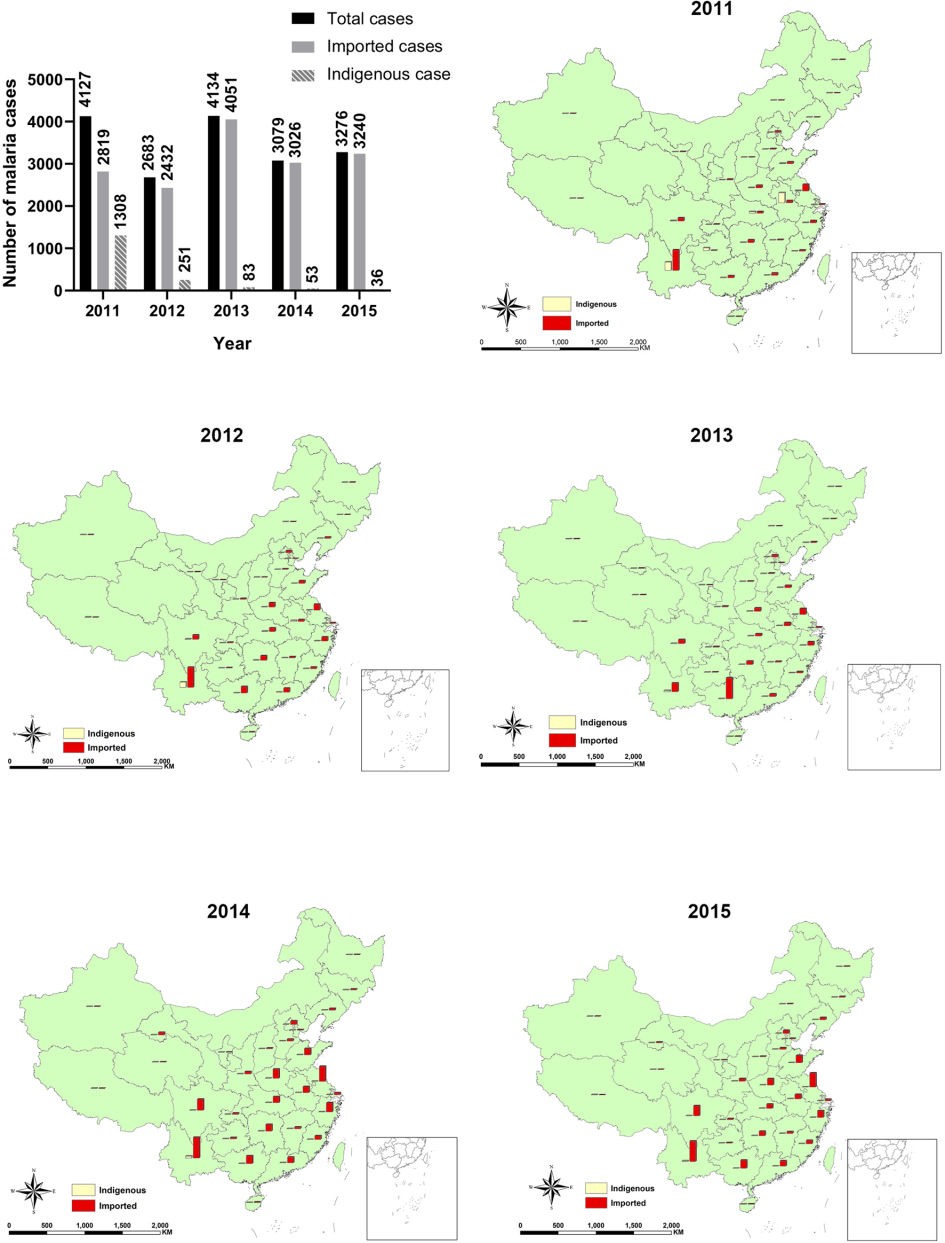
Number of malaria cases and their spatial distribution in China from 2011 to 2015. The number of indigenous cases has decreased year by year after the implementation of 1-3-7 approach

Although China has become a malaria-free country, imported malaria remains a threat. It was reported by Xia et al. that a total of 30,278 malaria cases were reported in China from 2011 to 2020, including 165 deaths. Of these cases, 1732 were indigenous and 28,173 were imported. There were still thousands of imported malaria cases in those year, but the number of indigenous cases has decreased year by year, from 1308 in 2011 to 36 in 2015 [15] (Fig. 3). The last indigenous case was reported in Yunnan Province in April 2016, and no indigenous cases have been reported since 2017 [9]. The timeliness of the 1-3-7 approach has greatly contributed to eliminating malaria in China. In addition, the large number of imported cases did not lead to transmission of malaria, also owning to the 1-3-7 approach [16]. A country can be awarded the certification of malaria-free status by WHO after it has been proved that local human malaria transmission has been interrupted for at least three consecutive years and a national surveillance system and a programme for the prevention of reintroduction are in place. On June 30, 2021, WHO certified China free of malaria [17]. The 1-3-7 approach played a very important role in this process.

### Successful experience of 1-3-7 approach in China

1-3-7 approach is a resource intensive and vertical approach. Therefore, it is more suitable to eliminate malaria transmission quickly in countries with low malaria transmission [18]. With the incidence by county remaining less than 1/10,000 in most Provinces in 2009, China transformed its strategy from controlling malaria to elimination [19]. Jiangsu Province is one of the provinces with the best compliance of 1-3-7 approach. Jiangsu Province successfully achieved zero indigenous malaria reports in 2012, a total of 2423 imported cases were reported in Jiangsu Province between 2012 and 2020. Cao et al. reported that 100% of these cases were reported through CISDCP within 24 h, more than 99.4% of the malaria cases were verified and received epidemiological investigation within 3 days, and more than 98.3% of foci completed investigation and response within 7 days [20]. Similarly, Shanxi Province and Henan Province, which achieved zero indigenous malaria since 2012, also have a well adherence to the timelines of 1-3-7 approach [21, 22].

In addition to epidemiological characteristics, capacity of health facility personnel also influence adherence to the timeliness of 1-3-7 approach. From 2011 to 2016, a malaria diagnosis reference laboratory network covering all 24 endemic Provinces was gradually established, strengthening the quality assurance of malaria detection and diagnosis nationwide [23]. Malaria at the Chinese border has become a major malaria concern in China, with Yunnan Province reporting the most imported cases. From 2011 to 2019, a total of 5254 malaria cases were reported in Yunnan Province, including 688 indigenous cases (13.1%) and 4,566 imported cases (86.9%) [16]. In the initial phase (2012–2014), the 1-3-7 approach was well executed, but the timeliness needed to be further strengthened [24]. About 81.5% of case investigation completed within 3 days. Of the reported cases, 70.0% had foci investigation and response, of which 90.1% were completed within 7 days [25]. Through training staff in case investigation and foci response, the timeliness of the 1-3-7 approach was improved. According to statistics, 95.6% of cases completed investigation within 3 days, 97.9% of foci were investigated and disposed within 7 days from 2013 to 2019 [26].

### Challenges of 1-3-7 approach in China

Although the 1-3-7 approach was well executed in China, case reporting, investigation and foci response was not entirely happening according to plan [27]. There were several challenges during the implementation. (1) The location of some reported cases in remote villages was the main reason, which resulted in CDC staff spending more time on transportation to complete case investigation and foci response. [28–30]. (2) Follow-up was difficulty in imported cases. The mobility of imported cases is complex, which increased the difficulty of investigation and sometimes even led to the loss of cases [31]. For example, Yunnan’s 4060 km long border without natural barriers leads to frequent population movements with surrounding countries, such as Laos, Myanmar and Vietnam [32]. (3) Poor awareness of healthcare-seeking led to delays in the implementation of 1-3-7 approach. Local poverty and illiteracy reduce access to health care [33, 34]. Wang et al. [21] collected 90 malaria cases in Shanxi Province from 2012 to 2018, and found that the average interval from onset to medical treatment was 3 days. (4) Some villages did not have the capacity to diagnose or notify malaria, resulting in the 1-3-7 approach not triggered in time. It has been shown that the median time between healthcare seeking and malaria diagnosis was 2 days in Shanxi Province, and this is also observed in Jiangsu Province [21, 35].

### Performance of 1-3-7 approach in other countries

The Great Mekong subregion (GMS) is the epidemic area of malaria. In 2021, the six GMS countries, China, Cambodia, Lao People’s Democratic Republic, Myanmar, Thailand and Viet Nam, reported 65,297 malaria cases [36]. China’s malaria-free status provides an important blueprint for the Greater Mekong region. As other GMS countries enter the final stages of malaria elimination, they have popularized and integrated 1-3-7 approach to surveillance systems that can accelerate the targets for malaria elimination. Thailand, Cambodia and Lao People’s Democratic Republic all follow the 1-3-7 approach. Myanmar has adjusted to a 1–7 approach, requiring cases reporting within 24 h, and foci investigation and response within 7 days. Viet Nam has changed to a 2–7 approach, requiring foci investigation within 2 days and foci response within 7 days [37].

After decades of malaria intervention in Thailand, most of the population was living in malaria-free areas [38]. As a result, Thailand changed its malaria control programme to malaria elimination programme in 2016, aiming to achieve malaria-free status by 2024. Since 2013, the number of active foci reported in Thailand has decreased year by year. But in 2017 there was an outbreak of malaria. Between May and July, monthly reported cases exceeded the epidemic threshold and reached a peak. During the outbreak, Thailand adopted 1-3-7 approach as part of regular malaria prevention and response approach, which played an important role in controlling the outbreak [39]. The timely implementation of 1-3-7 approach reduces the risk of malaria transmission in foci and reduces the probability of becoming active foci. As reported by Lertpiriyasuwat et al. [40], the number of malaria cases in Thailand decrease from 14,948 in 2017 to 4421 in 2019 after the implementation of 1-3-7 approach, with a 70% reduction in incidence. On the other hand, investment in malaria elimination surveillance has significantly reduced the burden on Thailand’s health system and economy. However, due to the lack of human resources at the corresponding level in Thailand, the compliance of 1-3-7 timeliness needs to be improved. Sudathip et al. [41] reported that foci with over 80% adherence to 1-3-7 timeliness approach reduced malaria risk by 22%. Therefore, improving the timeliness of 1-3-7 approach would help Thailand mitigate future outbreaks and achieve malaria elimination as soon as possible.

Cambodia aims to eliminate malaria by 2025 [42]. To achieve this goal, the 1-3-7 approach was scale-up in Sampov Loun in July 2015 [43]. As the implementation of 1-3-7 approach progressed, its timeliness improved significantly. The number of cases reported within one day increased from 50% in July 2015 to 100% in January 2017, case investigation rose from 20 to 100% within 3 days, and foci investigation and response within 7 days increased from 35% to nearly 100%. Correspondingly, the number of malaria cases decreased from 519 in 2015 to 181 in 2017 (incidence rate dropped from 3.21 per 1000 in 2015 to 1.06 per 1000 in 2017) [43]. This result also led to the implementation of 1-3-7 approach across Cambodia to achieve the goal of malaria elimination by 2025.

Malaria morbidity and mortality in Myanmar decreased significantly in recent year, but it remains a major public health problem in Myanmar [1]. In 2016, Myanmar adopted China’s 1-3-7 approach with the aim of achieving malaria elimination by 2030. Kyaw et al. [44] reported that in the initial phase of 1-3-7 approach in Myanmar, only 312 (32.5%) of the 959 patients completed case investigation and foci response. Poor knowledge of 1-3-7 approach among health workers was the challenge in early phase of implementing the approach, which could prevent the malaria elimination approach from reaching its full potential [45]. In addition, insufficient human resource availability and transportation difficulties also resulted in delay in 1-3-7 approach. Therefore, consideration must be made for locally tailoring in most malaria endemic settings across Myanmar.

The 1-3-7 approach has also been adopted in Africa such as Tanzania to achieve malaria control [10]. A pilot project in Rufiji District, southern Tanzania, from September 2015 to June 2018, showed the 1,7-malaria reactive community-based testing and response approach, which originated from the 1-3-7 approach, significantly reduced the malaria burden in the areas of high transmission [46].

### Challenges and limitations in the implementation of 1-3-7 approach globally

Even though countries have successfully implemented 1-3-7 approach, there have been several challenges and limitations. (1) The major reason is that 1-3-7 approach is a resource intensive and vertical approach. In countries with high malaria transmission, 1-3-7 approach is difficult to eliminate malaria transmission quickly because it is more labor- and time-consuming. RACD, a process of identifying secondary cases following the detection of a case, needs to be expanded to cover all residents in the foci. The type of RACD can be divided into by microscopy, RDT, and PCR [47]. Although the yield from PCR was four times higher than RDT and microscope, PCR screening was more expensive, labour- and time-consuming, and requires advanced equipment, making it not the preferred screening method [48]. In areas with high malaria transmission, RACD is not recommended as a strategy to malaria elimination because its resource intensive, while mass drug administration can have strong immediately effects [49]. Therefore, the 1-3-7 approach is more suitable for malaria elimination in areas where malaria is already under control [18]. The 1-3-7 approach needs to be tailored according to the region condition. (2) The reported cases occurred in remote areas also affected the implementation of 1-3-7 approach. For example, most of Myanmar’s population lived in rural areas, which undoubtedly made the timeliness of 1-3-7 approach more difficult. Kyaw et al. [44] reported that of the 959 malaria cases diagnosed in 2016, only 312 (32.5%) patients completed the 1-3-7 approach in time. (3) Insufficient knowledge of the health workers. Some health workers did not have the skill to diagnose malaria on blood smears or to identify malaria types to classify the foci types. On the other hand, the lack of understanding of 1-3-7 approach also affected the implementation of the approach [45].

### Suggestion for improving the performance of 1-3-7 approach

The period before “1” mentioned in the 1-3-7 approach refers to the interval from the symptom onset to diagnosis, which is not under the control of public health facilities. From 2012 to 2018, only 13.14% (170/1294) of malaria cases in Henan Province in China were diagnosed within 24 h after onset. The median time from onset to diagnosis was 3 days [22]. Similarity, Feng et al. reported that it took an average of 8.5 days for patients from onset to diagnosis in the China-Myanmar border region in 2013–2014 [24]. This is mainly due to patients’ low awareness of malaria diagnosis and treatment, and the lack of experience and capacities in some township hospitals for malaria diagnosis [50–52]. However, the interval from symptom onset to diagnosis cannot be ignored. Earlier diagnosis and treatment lead to better prognosis, lower mortality and the avoidance of transmission. Therefore, publicizing malaria knowledge is very important, especially for people returning home from or travelling abroad to malaria-endemic areas. At the same time, monitoring and follow-up should be strengthened for these high-risk groups [53]. Inadequate capacity of health workers can also lead to delays in diagnosis, so training health workers is of particular importance. Cao et al. [52] found that the knowledge of 1-3-7 approach improved significantly after the tabletop exercise module.

Poor surveillance system in some countries or regions makes it difficult to notify cases, which leads to a series of problems such as delayed treatment and increased risk of malaria transmission. For example, only about one-tenth of all malaria cases in India in 2013 were detected and reported [1]. In order to solve poor surveillance, Ethiopia, India and other countries use information technology and mobile apps to achieve the surveillance assistants [54, 55]. It was reported that these surveillance assistants can directly obtain basic information of outpatient cases and follow up patients to complete case investigation. On the other hand, the software can document foci information, so as to complete foci response more effectively.

The investigation and response of foci should not be relaxed. According to statistics, of the 101 non-active foci in China in 2015, only 81 (80.2%) of foci completed investigation and response within 7 days, which provided potential risk for malaria transmission [50].

A high number of cases are reported in China each year when most overseas workers return home for holidays [21]. Therefore, enhancing surveillance during this period is critical to improving the timeliness of the 1-3-7 approach. Moreover, public health awareness through health education should be enhanced and more plentiful health supplies and equipment need to be supported to the endemic area.

## Conclusion

Malaria has afflicted humanity for millennia. The implementation of tools and strategies today has saved many lives and even eliminated malaria in some country. The 1-3-7 approach proposed by China has played an important role in eliminating malaria. Although the implementation of 1-3-7 approach has significantly reduced the burden of malaria in many countries by preventing further spread of the malaria in a timely manner, the 1-3-7 approach needs to be tailored according to the region condition. Because it is more labour- and time-consuming, it is more suitable for countries where malaria is under control. For malaria in remote areas, travel constraints affect the timeliness of 1-3-7 approach. It is also very important to popularize malaria knowledge, train staff, improve the capacity of township health centres and monitor high-risk groups to improve the performance. Moreover, the 1-3-7 approach is a time request to the response, the implementation and completion of the response is also critical to interrupting malaria transmission and retransmission. After all, remaining vigilance in detecting malaria cases and optimizing surveillance and response systems are critical to achieving and sustaining malaria elimination.

## Data Availability

The data used in this report is available to readers.

## Abbreviations

WHO: World Health Organization
NMEAP: National Malaria Elimination Action Programme
FUO: Fever of unknown origin
CISDCP: China Information System for Diseases Control and Prevention
CDC: Center for disease control and prevention
GMS: Great Mekong subregion
RACD: Reactive case detection
