# Correction: 1-3-7 surveillance and response approach in malaria elimination: China’s practice and global adaptions

**DOI:** 10.1186/s12936-023-04608-0

**Published:** 2023-06-08

**Authors:** Boyu Yi, Li Zhang, Jianhai Yin, Shuisen Zhou, Zhigui Xia

**Affiliations:** National Institute of Parasitic Diseases, Chinese Center for Disease Control and Prevention (Chinese Center for Tropical Diseases Research), NHC Key Laboratory of Parasite and Vector Biology, WHO Collaborating Center for Tropical Diseases, National Center for International Research on Tropical Diseases, Shanghai, 200025 China

**Correction: Malaria Journal (2023) 22:152** 10.1186/s12936-023-04580-9

At the request of the authors, following publication of the original article [1], the map in Fig. 3 has been updated. The updated Fig. 3 is included in this erratum for reference.

**Fig. 3:**
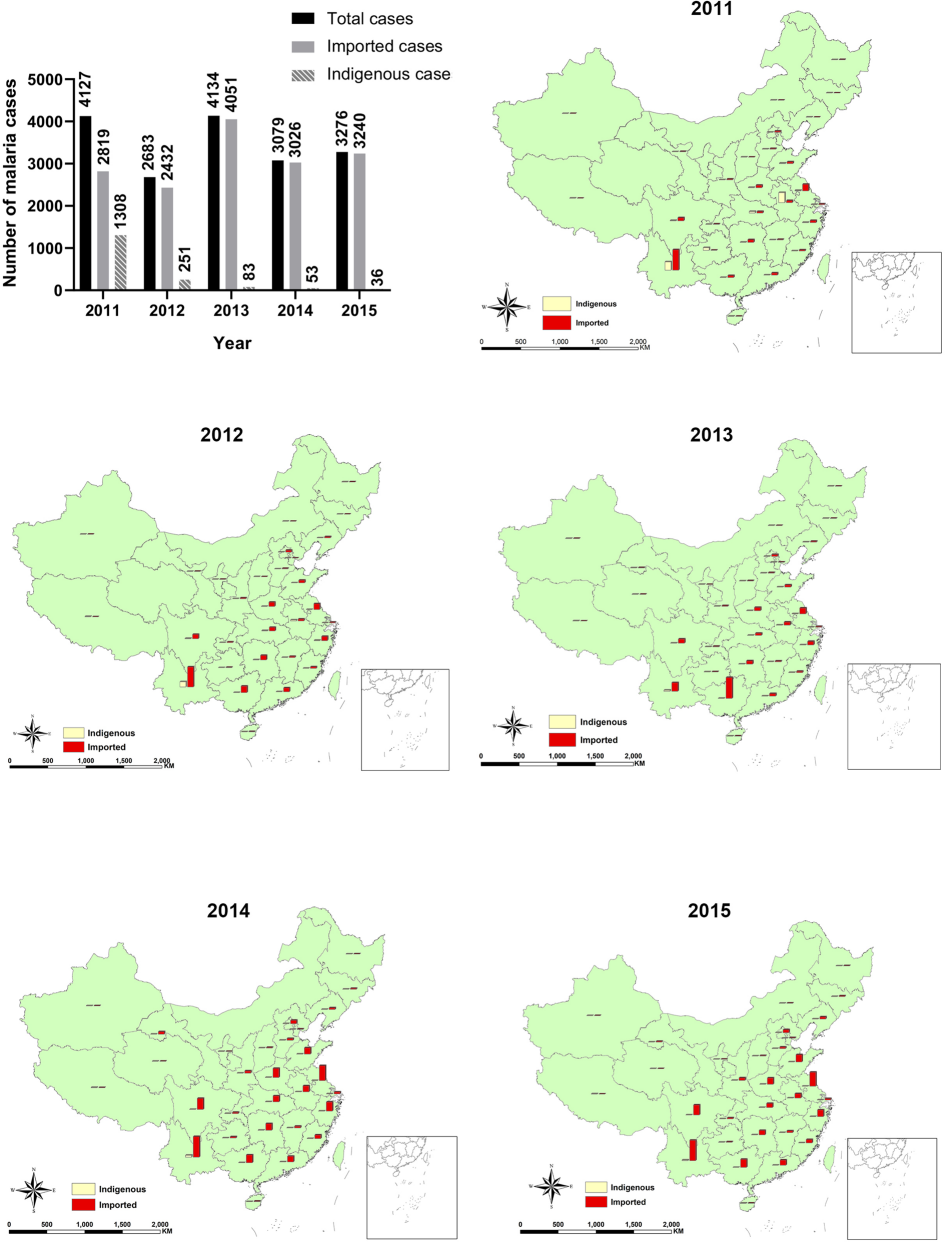
Number of malaria cases and their spatial distribution in China from 2011 to 2015. The number of indigenous cases has decreased year by year after the implementation of 1-3-7 approach

The authors would like to highlight that this change does not affect the statistics or conclusions of the article, and thank you for reading this erratum and apologize for any inconvenience caused.
